# Pairwise approach for analysis and reporting of child's free sugars intake from a birth cohort study

**DOI:** 10.1111/cdoe.12770

**Published:** 2022-07-11

**Authors:** Huy Van Nguyen, Diep Hong Ha, An Thi Minh Dao, Rebecca K. Golley, Jane A. Scott, John Spencer, Lucinda Bell, Gemma Devenish‐Coleman, Loc Giang Do

**Affiliations:** ^1^ Health Innovation and Transformation Centre Federation University Ballarat Victoria Australia; ^2^ School of Medicine and Dentistry Griffith University Gold Coast Queensland Australia; ^3^ Department of Population and Quantitative Health Sciences The University of Massachusetts Medical School Worcester Massachusetts USA; ^4^ School of Dentistry, Faculty of Health and Behavioural Sciences The University of Queensland Brisbane Queensland Australia; ^5^ Caring Futures Institute, College of Nursing and Health Sciences Flinders University Adelaide South Australia Australia; ^6^ School of Population Health Curtin University Perth Western Australia Australia; ^7^ Australian Research Centre for Population Oral Health University of Adelaide Adelaide South Australia Australia

**Keywords:** Australia, children, cohort studies, data analysis, dietary sugars, latent class analysis, methodological study

## Abstract

**Objectives:**

The prospective cohort design is an important research design, but a common challenge is missing data. The purpose of this study is to compare three approaches to managing missing data, the pairwise (*n* = 1386 children), the partial or modified pairwise (*n* = 1019) and the listwise (*n* = 546), to characterize the trajectories of children's free sugars intake (FSI) across early childhood.

**Methods:**

By applying the Group‐based Trajectory Model Technique to three waves of data collected from a prospective cohort study of South Australian children, this study examined the three approaches in managing missing data to validate and discuss children's FSI trajectories.

**Results:**

Each approach identified three distinct trajectories of child's FSI from 1 to 5 years of age: (1) ‘low and fast increasing’, (2) ‘moderate and increasing’ and (3) ‘high and increasing’. The trajectory memberships were consistent across the three approaches, and were for the pairwise scenario (1) 15.1%, (2) 68.3% and (3) 16.6%; the partial or modified pairwise (1) 15.9%, (2) 64.1% and (3) 20.0%; and the listwise (1) 14.9%, (2) 64.9% and (3) 20.2% of children.

**Conclusions:**

Given the comparability of the findings across the analytical approaches and the samples' characteristics between baseline and across different data collection waves, it is recommended that the pairwise approach be used in future analyses to optimize the sample size and statistical power when examining the relationship between FSI in the first years of life and health outcome such as dental caries.

## 1 INTRODUCTION

Excessive consumption of free sugars is among the leading causes of dental problems^1^, ^2^ and is also associated with other systemic health problems including obesity, diabetes and cardiovascular diseases.^3^, ^4^ In the field of dental health, like other disciplines, the most reliable design to investigate the association between free sugars intake and dental problems is a prospective cohort study.^2^ Longitudinal or cohort data allows researchers to evaluate temporal health problems, yet the analysis is likely hindered by complex unstructured, unbalanced mixtures of time‐varying and static covariate effects and missing data.^5^

One of the consequences associated with missing data is a reduction in statistical power. The other concern is related to the nature of missing data; whether it be missing completely at random (MCAR), missing at random (MAR) or missing not at random (MNAR), of those the latter pattern (MNAR) has a greater effect on findings.^6^ There are three common approaches to these challenges when managing missing data. The first approach is complete‐case analysis (also known as listwise deletion analysis) which is a common default option as most statistical software programs analyse data only from those participants without missing values. This method is recommended when missingness is MCAR; otherwise, it yields biased, less precise estimates.^5^ Another approach based on available data (known as pairwise deletion analysis) can help minimize the loss of data, yet at times, may lead to mathematically inconsistent results,^7^ especially when the sample is not large, or the missingness is MNAR.^8^

The third approach is the use of imputation methods—simple or multiple imputation. The simple method imputes missing values only once, and thus, disregards the uncertainty of the imputed values, thereby biasing standard errors, leading to artificially narrow confidence intervals that can give false precision.^8^ A remedy for this is multiple imputation to predict missing values from an appropriate stochastic model. Despite its advantages over single imputation, multiple imputation is not recommended for data with MNAR and when the proportions of missing data are very large (for example, more than 40%) on important variables,^9^ as results may only be considered as artificial or hypothesis‐generating results.^10^ Nevertheless, biases caused by MNAR can be addressed by sensitivity analyses examining the effect of different assumptions about, and/or approaches to, missing data.^11^ While there is a great deal known about these methods, there remains an important absence of research using a partial or modified pairwise deletion analysis. Using this method as an additional option enhances validation of the research findings which in turn informs analytical selection for addressing research objectives and hypotheses.

Statistical methods of analysing longitudinal data can be standard‐ or modern‐based and are categorized into three classes of commonly used approaches. The first class uses the summary statistic approach to condense the repeatedly measured information into a single number per subject but eliminating within‐subject repeated measurements to allow for a straightforward comparison of groups using standard statistical hypothesis tests.^12^ The second class comprises standard growth analyses such as the univariate and multivariate repeated‐measures analysis of variance (also known as ANOVA and MANOVA, respectively) or structural equation modelling (SEM), allowing for comparing group means (e.g., the scores between ‘time 1’, ‘time 2’ and ‘time 3’, or more categories). Neither of these approaches assess subject‐specific trends over time, strong assumptions are rarely met and low flexibility with regards to missing data limits the use of this approach.^12^, ^13^ To address these problems, the third modern‐based class of analysis uses flexible regression‐based techniques.^12^ One such approach is group‐based trajectory modelling (GBTM) known as latent class growth modelling. This model that we aim to apply can fit missing data using maximum likelihood estimation if the data are MNAR.^14^

Group‐Based Trajectory Modelling (GBTM) was first introduced by Nagin and Land in 1993^15^ as a statistical method for analysing developmental trajectories of any outcome over time.^16^ Since then, it has been extensively applied in a wide range of disciplines.^16^ Recently there has been a rapid rise in its use in clinical research^14^, ^17^ and it is a useful method for statistical modelling to inform policy and practice.^16^ The field of oral health, however, has witnessed a limited use of this method.^18^ Longitudinal research investigating development of free sugars intake (FSI) advances understanding of behaviour and informs early and timely public health preventions. There are, however, few cohort studies that follow‐up the effect of sugar‐related feeding practices on dental health^19^, ^20^ and use GBTM.^14^ Using GBTM as a main statistical model, this study examined three approaches to managing missing data to characterize children's FSI trajectories during the first 5 years of life using data from the Study of Mothers' and Infants' Life Events Affecting Oral Health (SMILE). As GBTM works with missing data,^17^, ^21^ it supports the examination of three analytical approaches to missing data; (1) the pairwise, (2) the partial pairwise and (3) the listwise.

## 2 METHODS

### 2.1 Study design and ethics

The SMILE cohort is a population‐based cohort of mothers and their children from Adelaide, Australia. A detailed description of the study protocol and the study cohort have been published elsewhere.^22^, ^23^ Data for the present analyses were collected at recruitment (baseline) and from three waves (2014–2015, 2015–2016 and 2018–2019) of the SMILE study, when the children turned 1, 2 and 5 years of age, respectively. Ethical approval for SMILE was obtained from relevant Human Research Ethics Committees across South Australia, HREC#50.1 (28/02/2013), HREC#13/WCHN/69 (07/08/2013) and HREC#H‐2018‐017 (16/10/2018). All participants agreed to participate and provided informed consent.

### 2.2 Group‐based trajectory model

Capitalizing on recent advances in GBTM, this study adopted the GBTM technique to identify FSI trajectories of the children over the course of 5‐years follow‐up. The methodology of this technique has been fully described in Supplement.

### 2.3 Measurement of key variables and indicators

#### 2.3.1 Child's Free Sugars Intake (FSI)

Child's usual FSI (grams per day) was estimated at each of the three waves. In wave 1 (2014–2015, when children had turned 1 year old), 3 days of non‐consecutive dietary intake data were collected for each child via a 24‐hr recall and 2‐day food record, entered into FoodWorks version 8 (Xyris Software, 2012–2017), and FSI estimated using the Australian food composition database, AUSNUT 2011–13.^24^, ^25^ In waves 2 (2015–2016, 2 years of age) and 3 (2018–2019, 5 years of age), FSI was estimated using a Food Frequency Questionnaire (FFQ) that was designed for use with this cohort.^25^ The SMILE FFQ consisted of a food list of 89 items primarily identified as being major food and beverage sources of total and free sugars. Frequency and quantity response options appropriate to this age group were developed for each item. Seven frequency response options were used for all items, commencing with ‘never or rarely’ before ranging from ‘1 time every 2 weeks’ to ‘3 or more times per day’. Quantity response options were tailored to each item, comprising household measures (teaspoon, tablespoon, cup) or typical portion sizes (piece, tub, pouch etc.). Finally, a database was developed to analyse the SMILE‐FFQ, linking scoring algorithms for all possible frequency responses to grams of total and free sugars, derived from representative foods in the AUSNUT 2011–13 food composition database.^24^ Validation of the FFQ indicate that estimates of FSI derived by 24‐hour recalls and FFQ are comparable.^25^ These variables were then used for the GBTM to investigate children's FSI trajectories.

#### 2.3.2 Socio‐demographic characteristics

The socio‐demographic variables comprised the mother's age at birth (years), mother's education attainment (High School, Vocational Training, Tertiary), Index of Relative Socio‐Economic Advantage and Disadvantage for Areas (IRSAD decile, from 1 (most disadvantaged) to 10 (most advantaged)), mother's country of birth (Australia and New Zealand vs others), household income (4 levels, from 1 as Lowest to 4 as Highest) and parent household type (single‐, two‐parent household), child's sex (female, male) and number of children in the household (1, 2, ≥3) reported at baseline (child birth). These served as key variables for profiling child's FSI trajectories.

### 2.4 Statistical analysis

As is common for longitudinal studies, child FSI variables were missing across different waves of the study. As the proportion of missing data at three waves was 46.38%, 51.56% and 66.41%, respectively, multiple imputation was not used as recommended by literature.^10^ To address the challenges of panel data with missing values, instead of conducting conventional analyses with either available‐case or complete‐case, three analytical approaches were conducted: (1) pairwise deletion (available‐case), (2) partial pairwise deletion and (3) listwise deletion (complete‐case). Pairwise deletion analysis k complete cases were used as many as possible, meaning that any cases with child's FSI variable from at least one wave were included. In partial pairwise analysis, cases with at least two waves of child's FSI variables completed were included. The listwise deletion method involved the analysis of only cases with complete child's FSI variables from all 3 waves. These analytical methods help to assess the level of consistency of results and the method that best describes the data. The total samples equivalent to these analytical methods were 1386, 1019 and 546 mother–child dyads, respectively. The consistency of the results across the methods was also evaluated by graphs and parameters and *t*‐, *F*‐ and χ^2^‐test statistics.

Prior to GBTM, three waves of data on both mothers and children were merged. The data on mothers contained socio‐demographic information, while the data on children comprised child's FSI variables from the three study waves. As child's FSI variables were right skewed which could affect the precision of modelling, these variables were log‐transformed so they became normally distributed (Figure S1), and then GBTM was performed to generate child's FSI trajectories as described in the Supplement. The three items of child's FSI from the three waves of the SMILE study served as essential inputs for GBTM to identify a new latent variable, *child's FSI trajectory group*, which included three subgroups of child's FSI trajectories as identified by the GBTM below.

Following the technique of GBTM, the first step was to identify the number of child's FSI trajectories. As the quadratic component of the single quadratic model was significant (*p* < .001, Table S1, Figure S2), the higher trajectory quadratic modelling was performed (Tables S2–S4; Figures S3–S5). A comparison of BIC (Bayesian information criterion) and the log Bayes factor across the single‐, two‐, three‐ and four‐trajectory quadratic models of three waves of child's FSI was summarized in Table S6. Although the quadratic component of the four‐trajectory quadratic model was significant (Table S4), this model was not selected for four reasons, (1) its BIC is higher than the three‐trajectory quadratic (−4117.20 vs. −4147.97, Table S6), (2) the membership probability of the first group <5% (Table S4),^13^ 3) the wide 95% CI^16^, ^26^ and (4) the similar (parallel) pattern of group 3 and 4 suggested they should be combined (Figure S5). As a result, a three‐trajectory quadratic model was adopted for the next steps.

Although the three‐trajectory model was relatively fit, the simplification process based on a comparison with a two‐quadratic and one‐linear model was conducted. First, as seen from Table S6, even though both models showed strong evidence (log Bayes factor >2), the two‐quadratic and one‐linear model had a lower BIC (−4166.15 vs. −4147.97). Second, close to 60% of the child population with high increasing FSI in the three‐quadratic model is not practical (Figure S4). The other fit criteria from Table S7 showed that the two‐quadratic and one‐linear model is of better fit than the three‐quadratic model, such as the narrower 95%CI of the group membership probabilities, higher average posterior probabilities, higher weighted odds of correct classification (Table S7). For these reasons, the two‐quadratic and one‐linear model was chosen.

All analyses were performed in Stata version 17. Statistical tests were 2‐sided with a significance level of *p* < .05 set.

## 3 RESULTS

The key characteristics of samples of mothers participating in at least one of three waves are displayed in Table 1. The characteristics of child participants are presented in three samples, corresponding to samples for the three methods of analysis: the pairwise, partial pairwise and listwise deletion, *n* = 1386, *n* = 1019 and *n* = 546, respectively. Despite different sample sizes, the samples were comparable with regard to key background variables. The mean age of mothers in all samples was slightly over 30 years. The majority were Australians and New Zealanders, with two‐parent households, tertiary educated or had incomes of >$AUD40,000 to <120 000 per year. Approximately 50% of the mothers had one child, with an approximately even split/distribution of boys and girls. The mothers in the listwise deletion sample were, however, more likely to be tertiary educated and an income of >$120 000 per year than the baseline sample (62.2% vs. 52.7% and 26.3% vs. 22.0%, respectively). The mean of the IRSAD decile among the listwise deletion sample was also somewhat higher than among the baseline sample (5.9 vs. 5.5). However, the differences are minimal.

**TABLE 1:** Characteristics of the samples mothers and children were merged. The datas per three different methods of analysis^a^

Characteristics	Mother sample (baseline)	Child sample 1^b^	Child sample 2^c^	Child sample 3^d^
	*N* = 2182 *n* (%)	*N* = 1386 *n* (%)	*N* = 1019 *n* (%)	*N* = 546 *n* (%)
Mother's age (years)	30.3 (5.2)	30.4 (5.1)	30.6 (5.0)	31.0 (5.0)
IRSAD decile, mean (SD)	5.5 (2.8)	5.6 (2.8)	5.7 (2.8)	5.9 (2.8)
Mother's highest education level				
High school	312 (21.2)	248 (19.2)	168 (16.6)	72 (13.3)
Vocational training	383 (26.1)	344 (26.6)	251 (24.8)	133 (24.5)
Tertiary education	775 (52.7)	703 (54.3)	594 (58.6)	337 (62.2)
Mother's country of birth				
Others	465 (31.3)	386 (29.5)	279 (27.4)	149 (27.3)
Australia and New Zealand	1023 (68.8)	921 (70.5)	740 (72.6)	397 (72.7)
Household income ($A)				
≤40 000	216 (15.3)	165 (13.3)	113 (11.6)	51 (9.7)
>40 000–80 000	465 (33.0)	407 (32.8)	313 (32.0)	160 (30.3)
>80 000–120 000	419 (29.7)	383 (30.8)	309 (31.6)	178 (33.7)
>120 000	310 (22.0)	287 (23.1)	243 (24.8)	139 (26.3)
Parent household				
Single‐parent household	96 (6.5)	74 (5.7)	55 (5.4)	28 (5.1)
Two‐parent household	1373 (93.5)	1221 (94.3)	960 (94.6)	516 (94.9)
No of children				
1	928 (45.2)	626 (46.8)	463 (46.7)	266 (50.2)
2	740 (36.0)	497 (37.1)	369 (37.2)	192 (36.2)
≥3	386 (18.8)	215 (16.1)	159 (16.0)	72 (13.6)
Child sex				
Female	1031 (47.3)	642 (46.3)	474 (46.5)	243 (44.5)
Male	1151 (52.7)	744 (53.7)	545 (53.5)	303 (55.5)

^a^ All data presented as *n* (%) unless otherwise stated.

^b^ Pairwise deletion analysis method.

^c^ Partial pairwise deletion analysis method.

^d^ Listwise deletion analysis method.

Table 2 displays summary statistics of child FSI across the three waves by different methods of analysis. Based on the pairwise deletion sample, there was an increasing non‐linear trend in FSI over time (*p*_‐Mann‐Kendall trend_ <.001). The mean FSI of the one‐year‐old children (wave 1) was 8.8 g versus 32.2 g for the two‐year‐old children (wave 2) (>3‐fold), and 44.2 g among the five‐year‐old children (wave 3). The same trend was also observed for the partial pairwise and listwise deletion sample. Mean FSI of a particular wave was also similar across the three approaches (*p* > .05).

**TABLE 2:** A comparison of child's free sugars intake as per three different methods of analysis and standard growth analysis

Child's FSI (grams per day)	N = 1386^a^			*N* = 1019^b^			*N* = 546^c^			*p*‐Value^d^
	Mean (SD)	Median	[Min, Max]	Mean (SD)	Median	[Min, Max]	Mean (SD)	Median	[Min, Max]	
Wave 1, One‐year‐old	8.8 (12.0)	5.8	[0.0, 183.4]	8.7 (12.4)	5.7	[0.0, 183.4]	8.9 (14.3)	5.5	[0.0, 183.4]	.956
Wave 2, Two‐year‐old	32.2 (37.8)	23.0	[23.0, 540.5]	30.0 (31.6)	22.0	[0.3, 487.2]	29.0 (30.8)	21.1	[1.2, 487.2]	.116
Wave 3, Five‐year‐old	44.2 (45.7)	31.9	[2.1, 560.0]	43.2 (44.2)	31.5	[2.1, 560.0]	43.2 (46.4)	30.7	[2.7, 560.0]	.829
*p*‐Value^e^	<.001			<.001			<.001			

^a^ Pairwise deletion analysis method.

^b^ Partial pairwise deletion analysis method.

^c^ Listwise deletion analysis method.

^d^ *p*‐value based on the ANOVA F‐statistics.

^e^ *p*‐value based on a standard growth analysis, the Mann‐Kendall trend statistics.

**FIGURE 1:**
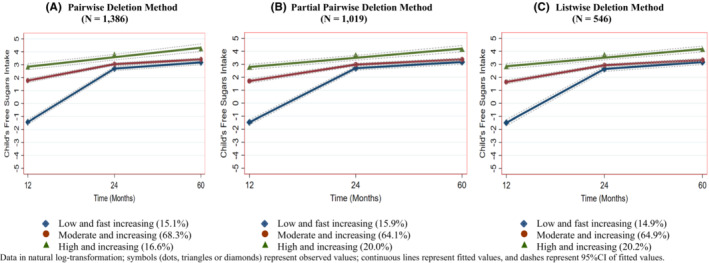
Trajectories of child's free sugars intake as per three different methods of analysis

Figure 1 plots the trajectories of child's FSI identified from GBTM using the censored normal model. The analysis achieved the optimal fit using a three‐group model, in which, for the pairwise deletion sample for instance, 15.1% of children were identified into a ‘low and fast increasing’ trajectory (group 1), 68.3% were grouped into a ‘moderate and increasing’ trajectory (group 2), and the remaining 16.6% were assigned to the ‘high and increasing’ trajectory (group 3). At age 1 year (wave 1), children in group 1 displayed an initial low level of FSI, a fast increase during the second year, and from there through year 5 a slow increase. At year 1, children in group 2 demonstrated a moderate FSI initially, and then a steady increase during the second year, and a slightly slower increase from year 2 through year 5. Group 3 showed a consistent linear trend from year 1 through year 5, with a high FSI at the beginning. However, regardless of the group, from year 2, all groups showed a remarkably similar trend. For the partial pairwise and listwise sample, just a slight difference from the pairwise was observed where more than 64% and about 20% of children were classified as having ‘moderate and increasing’ and ‘high and increasing’ trajectories, respectively. Despite this difference, the same trend of FSI was also found across the samples.

Table 3 reports profiles of the selected socio‐demographic characteristics of children following the three FSI trajectories shown in Figure 1. For the pairwise method of analysis, children in the ‘low and fast increasing’ group tended to have the mothers who were older, more educated, higher‐income earners, living in a postcode with the highest IRSAD score, of dual‐parent household and had two or fewer children. By contrast, children in the ‘high and increasing’ were least likely to hold these characteristics. In between are the profiles of the ‘moderate and increasing’ group. The same results were also observed for the remaining methods of analysis, the partial pairwise and the listwise.

**TABLE 3:** Child's free sugars intake trajectory group profiles, by selected mother's and child's socio‐demographic characteristics^a^

Characteristics	*N* = 1386^b^				*N* = 1019^c^				*N* = 546^d^			
	Group 1	Group 2	Group 3	*p*‐Value	Group 1	Group 2	Group 3	*p*‐Value	Group 1	Group 2	Group 3	*p*‐Value
*N* %	165%	1095%	126%		136%	730%	153%		75%	377%	94%	
Mother's age (years, (SD))	30.8 (4.7)	30.6 (5.1)	28.5 (5.7)	<.001	30.9 (4.3)	30.9 (4.90)	29.2 (5.5)	<.001	31.08 (4.1)	31.40 (5.0)	29.1 (5.5)	<.001
IRSAD Decile, mean (SD)	6.2 (2.9)	5.6 (2.8)	4.6 (2.8)	<.001	6.2 (2.8)	5.8 (2.7)	5.0 (2.9)	<.001	6.68 (2.7)	6.02 (2.7)	5.0 (2.9)	<.001
Mother's highest education level												
High school	14.6	18.2	33.3	.001	12.5	15.6	24.8	.019	12.0	11.8	20.2	.130
Vocational training	27.4	26.8	23.1		27.9	23.9	26.1		29.3	23.1	26.6	
Tertiary education	58.0	54.9	43.6		59.6	60.5	49.0		58.7	65.1	53.2	
Mother's country of birth												
Others	33.8	28.9	29.7	.460	32.4	26.8	25.5	.360	34.7	26.5	24.5	.280
Australia and New Zealand	66.2	71.1	70.3		67.6	73.2	74.5		65.3	73.5	75.5	
Household income ($A)												
≤40 000	7.8	12.5	27.2	<.001	5.3	9.8	25.7	<.001	5.4	6.9	23.7	<.001
>40 000–80 000	31.4	32.4	37.7		31.6	32.6	29.7		28.4	29.4	35.5	
>80 000–120 000	37.9	30.9	21.1		39.1	31.7	24.3		41.9	34.9	22.6	
>120 000	22.9	24.2	14.0		24.1	26.0	20.3		24.3	28.8	18.3	
Parent household												
Single‐parent	3.2	5.6	10.3	.039	3.0	5.1	9.2	.049	4.1	4.8	7.4	.520
Two‐parent	96.8	94.4	89.7		97.0	94.9	90.8		95.2	95.2	92.6	
No of children												
1	51.6	46.0	47.1	.070	53.5	45.2	48.3	.093	59.5	48.1	51.1	<.001
2	35.7	38.2	29.4		34.1	39.2	30.6		36.5	39.1	24.4	
≥3	12.7	15.7	23.5		12.4	15.7	21.1		4.1	12.8	24.4	
Child sex												
Female	50.3	46.7	38.1	.100	50.7	46.7	41.8	.310	49.3	46.2	34.0	.071
Male	49.7	53.3	61.9		49.3	53.3	58.2		50.7	53.8	66.0	

*Note*: Group 1: Lower fast increasing trajectory; Group 2: Moderate increasing trajectory; Group 3: Higher increasing trajectory.

^a^ All data presented as *n* (%) unless otherwise stated.

^b^ Pairwise deletion analysis method.

^c^ Partial pairwise deletion analysis method.

^d^ Listwise deletion analysis method.

## 4 DISCUSSION

Based on a 5‐year longitudinal study, the current study examined three approaches to managing missing data to investigate the development of FSI trajectories among children in early childhood using GBTM, a latent class growth analysis. The technique was useful in this study to identify distinct subgroups of individuals following a similar pattern of change over time for FSI. Three distinct trajectories of child's FSI were identified, defined as being ‘low and fast increasing’, ‘moderate and increasing’ and ‘high and increasing’ FSI during the follow‐up period.

Although each individual may have a unique developmental course, individual variations in change can be summarized by a finite set of polynomial functions, each of which represents a discrete trajectory.^26^ As with any longitudinal research, this study suffers from missing data for the child's FSI, in all three waves. Given that missing data were considerable, data imputation is not recommended.^9^ Instead, the analysis was conducted as per three analytic methods with two assumptions about missing data. The first assumption is that data were missing at random, meaning that there might be systematic differences between the missing values and the observed values, but these differences can be explained by differences in certain observed covariates. In this case, as suggested by Sterne et al.^11^ the analysis should be pursued with inclusion of incomplete data. Following Sterne's recommendation, the results analysed as per pairwise deletion method (Panel A, Figure 1) was best choice as this method allowed most of children in the study even if FSI data were missing from two waves of the study were included.

The other assumption was the case of data missing not at random. To address this assumption, additional analyses were conducted as per two more scenarios associated with smaller samples, the partial pairwise and the listwise. A pattern of child's FSI trajectories corresponding to that of the pairwise deletion method was detected. Both partial pairwise and listwise deletion analysis showed that children were classified into three groups, (1) ‘low and fast increasing’, (2) ‘moderate and increasing’ and (3) ‘high and increasing’, with just minor differences in group membership. These results suggested that the pattern of child's FSI trajectories was consistent across different methods of analysis. The consistence of the findings of the child's FSI trajectories across the different analysis methods and the similarity in characteristics of the three samples of children suggest that data are missing at random. This result in turn underpins the first assumption about missing data for utilization of GBTM. This comprehensive analysis of the three methods has confirmed consistency of the observed trajectories of FSI among children. To our knowledge, there has been no other investigation of FSI trajectories in early childhood, with only one study by Peres et al. having examined sugar total consumption between ages of 6 and 18 years.^2^ Their study identified that <20%, 40% and > 40% of the children were classified as ‘high’, ‘upward’ and low sugar consumers, respectively. This difference is understandable given that two studies differ in ages of participants and measures of sugar intake.

Another way to look at data is to examine the observed FSI means of children across the waves of the study. It was found that the means changed between waves in a consistent manner, with only slight differences between the three analytic methods. This result follows the same pattern that was identified from the GBTM highlighting increasing trends of FSI. Overall, the mean FSI was just under 9, around 30 and over 43 g per day in wave 1, 2 and 3, respectively. These intakes were lower than that reported for similarly aged US children aged 2–5 years^27^ based on a standard growth analysis which averaged the individual trajectories of all children in the research sample. This single trajectory method is useful for studying research questions assuming that all individuals in a given sample are expected to change in the same direction across time with only the degree of change varying between people.^13^ According to Nagin,^28^ however, some health and psychological issues may follow a multinomial pattern in which both the strength and direction of change vary between people. It is not always the case to assume that all people from a sample would have the same pattern of change as seen from Figure S7. It lends support for the application of GBTM in investigating the development of FSI trajectories among children in early childhood.

As a longitudinal design based on a population‐representative sample,^23^ the study findings can be generalized to populations with similar socio‐economic characteristics. Using both standard growth and latent class growth analysis, this study can provide insights to look at sugar intake trend data and inform oral public health policy and practice. Using GBTM, it is possible to identify a number of distinct trajectories of FSI, especially when data on group trajectories cannot be directly observed. Despite these premises, the current study has limitations. Similar to other longitudinal studies, the study sample was affected by attrition.^23^, ^29^ This issue as part of data missing values has been addressed throughout the article using the three methods of analysis.

## 5 CONCLUSIONS AND IMPLICATIONS

This study demonstrated the applicability of the GBTM under different scenarios in analysing longitudinal data when data imputation is not appropriate. It was found that the results across different scenarios and methods of analysis are comparable in terms of sample characteristics, mean estimates, development of child's FSI trajectories, and profiles of child's FSI trajectory groups. In studies such as this, when consistency of findings has been achieved, there is a strong recommendation for using maximum available data to perform analysis as it improves the technical aspects of the study such as sample size and study power in planned analyses examining the relationship between FSI in the first years of life and dental caries and other health outcomes.

## CONFLICT OF INTEREST

We declare that we have no competing interest.

## Supporting information


**Appendix S1** Supplementary Information

## Data Availability

The data that support the findings of this study are available on request from the corresponding author. The data are not publicly available due to privacy or ethical restrictions.
