# Supplementary material for: Pairwise approach for analysis and reporting of child's free sugars intake from a birth cohort study
Source: Community Dent Oral Epidemiol. 2022 Jul 11;51(5):820–8. doi: 10.1111/cdoe.12770 (PMC10946696; doi:10.1111/cdoe.12770)
Supplement: Supplementary file 1 — Appendix S1 Supplementary Information [file CDOE-51-820-s001.docx]

# SUPPLEMENT

**Group-based Trajectory Model**

The key concept in this method is the distribution of an outcome conditional on age (or time); in this instance, the distribution of a child’s FSI trajectory denoted by *P*(*Y_i_*| *Age_i_*), where vector *Y_i_* represents individual *i*’s longitudinal sequence of behavioral outcome, child’s FSI, and Age*_i_* represents individual *i*’s age when each of those measurements is observed between 2014-2015, 2015-2016, and 2018-2019 when SMILE participants were followed-up. The GBTM assumes that the population distribution of trajectories arises from a finite mixture of unknown order *J*. The likelihood for each individual *i*, conditional on the number of groups *J*, could be written as:

*P*(*Y_i_*| *Age_i_*) = $\sum_{j=1}^{J} \pi^{j}$. *P*(*Y_i_*| *Age_i_, j*; $\beta^{j}$), (1)

where *π^j^* is the probability of membership in group *j*, and the conditional distribution of *Y_i_* given membership in *j* is identified by the unknown parameter vector which determines the shape of the group trajectory. For given *j*, conditional independence is assumed for the sequential realisations of the elements of *Y_i_*, *y_it_*, over the T periods of measurement. As a result, it is possible to write:

*P*(*Y_i_*| *Age_i_, j*; $\beta^{j}$) = $\prod_{t=i}^{T} P\left( Y_{it} \right|{Age}_{it}, j; \beta^{j}$), (2)

where *p*(.) is the distribution of *y_it_* conditional on membership in group *j* and the age of individual *i* at time *t*. Researchers used Tobit model to specify *p*(.) for the outcome, child’s FSI, with the censored normal distribution, which was designed for analyzing repeatedly measured scales of child’s FSI censored by either a scale minimum or maximum or both values. P(.) is assumed to follow a censored normal distribution to allow for possibly clustering at the minimum and maximum [[1](#_ENREF_1)].

The values of *π^j^* (*j* = 1, 2, 3, ..., *J*) are estimated by a multinomial logit function as:

*π^j^* = $e^{\theta}$*^j^ /* $\sum_{j=1}^{J} e$*^θj^,* (3)

where the parameters to be estimated, *θ_j_*, can take on any value provided that each of the resulting probabilities (*π^j^*) properly lies between 0 and 1, and the values of *π^j^* sum to 1 across the *J* trajectory groups [[1](#_ENREF_1)]. The link between time or age and the variable of our interest such as child’s FSI is modeled as a polynomial relationship; and the statistical program that estimates these models allows up for up to a quartic relationship. Take our data, for instance, when our child’s FSI, *y_i_,* is a continuous variable, the linkage is established via latent variable $y_{i}^{*j}$ as:

$y_{i}^{*j}$= $\beta_{0}^{j}$ +$\beta_{1}^{j}{Age}_{i}$*+*$\beta_{2}^{j}$ ${Age}_{i}^{2}$*+*$\beta_{3}^{j}$ ${Age}_{i}^{3}$*+* $\beta_{4}^{j}$ ${Age}_{i}^{4}$*+*$\varepsilon$*_i_,*  (4)

where $\beta_{0}^{j}$, $\beta_{1}^{j}$*,*$\beta_{2}^{j}$*,* $\beta_{3}^{j}$*and* $\beta_{4}^{j}$are parameters that determine the intercept and slope of the *j*^th^ trajectory, and where error term $\varepsilon$*_i_* is assumed to be of normal distribution with a mean of zero and constant variance *σ*^2^ [[1](#_ENREF_1)]. The parameter estimates are allowed to vary freely across the j groups, a feature that enables various groups of children to have distinctive trajectories of FSI.

Upon the model fit conducted, the model parameter estimates can be used to generate a child’s posterior probability of group membership, denoted $\hat{P}$(*j*| *Y_i_*). The posterior probability stores the likelihood that a child with the observed sequence of measurements *Y* belongs to trajectory group *j*. The posterior probability is used for assigning children to their most likely trajectory group and for assessing the precision with which the model fits the data. The proper formularisation for this purpose, as developed by Nagin in 2005 [[1](#_ENREF_1)], is:

| $\hat{P}$(*j*\| *Y_i_*) = | $\hat{P}$(*Y_i_*\| *j*)$\hat{\pi}$*_j_* | (5) |
| --- | --- | --- |
|  | $\sum_{j}^{J} \hat{P}(Y_{i}\vert j)\hat{\pi}$*_j_* |  |

Where $\hat{P}$(*Y_i_*| *j*) and $\hat{\pi}$*_j_* are calculated based on formula 2 and 3, respectively.

Using all procedures presented above, researchers subsequently produced the individual-level estimates for formulas 1-5 based on child’s FSI values obtained from different waves of SMILE. All model estimates were undertaken using maximum likelihood, where the maximisation was performed using a general quasi-Newton procedure. In this article, to detect the most fit model, we conducted modeling in two steps.

To identify the number of trajectories, researchers applied a general rule by Andruff et al. [[2](#_ENREF_2)]. For data sets with three time points, a single quadratic trajectory model is tested first. If the quadratic component of this model is not significant, the model for one linear trajectory is run to determine the BIC (Bayesian Information Criterion) value for this model. If the quadratic component of the model for one trajectory is significant, the analysis for the two-trajectory quadratic model is performed. This process was repeated with an increasing number of trajectories until the model of best fit was obtained, as determined by comparing the BIC values (the lower the better model) and the log Bayes factor estimated as 2 x (BIC*_larger model_* - BIC*_smaller model_*) [the higher the better model]. As recommended by Jones et al. [[3](#_ENREF_3)], values of 0 to 2 are interpreted as weak evidence for the more complex model, >2 to 6 are interpreted as moderate evidence, >6 to 10 are interpreted as strong evidence, and more than 10 are interpreted as very strong evidence.

**Supplementary figure 1. Distribution of child’s free sugars intake across three waves of measurement before and after log-transformation**

| 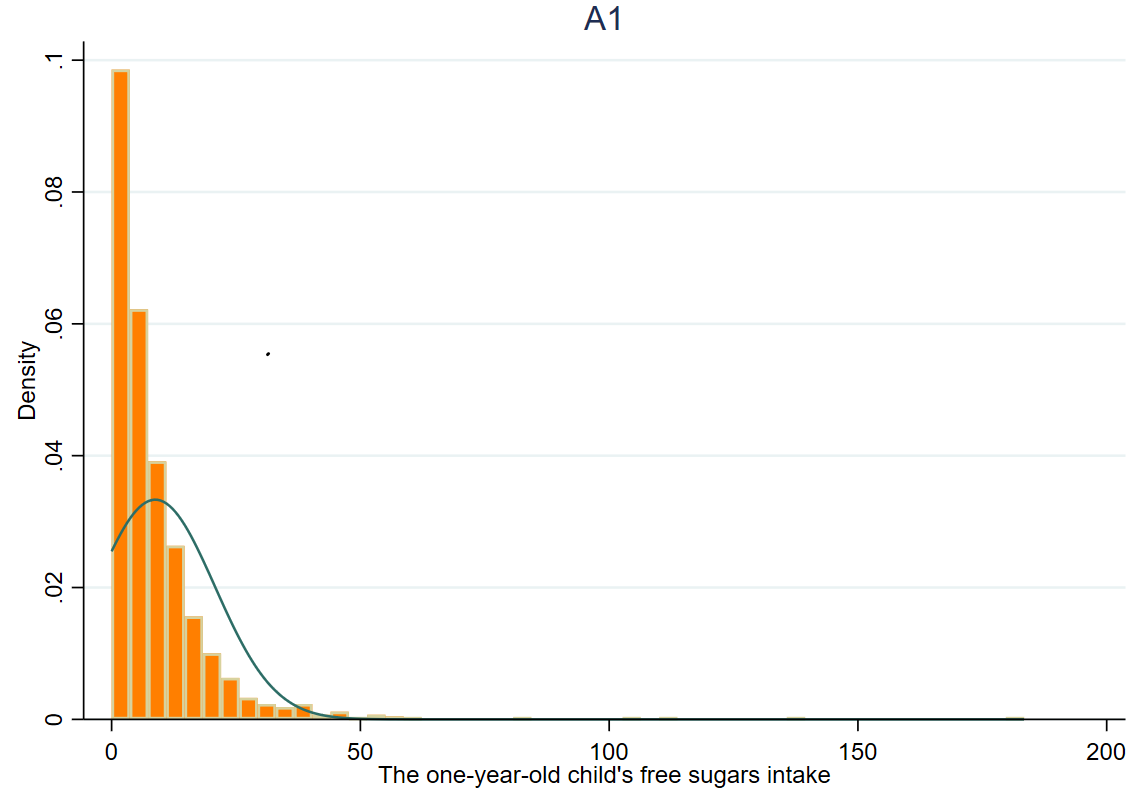 | 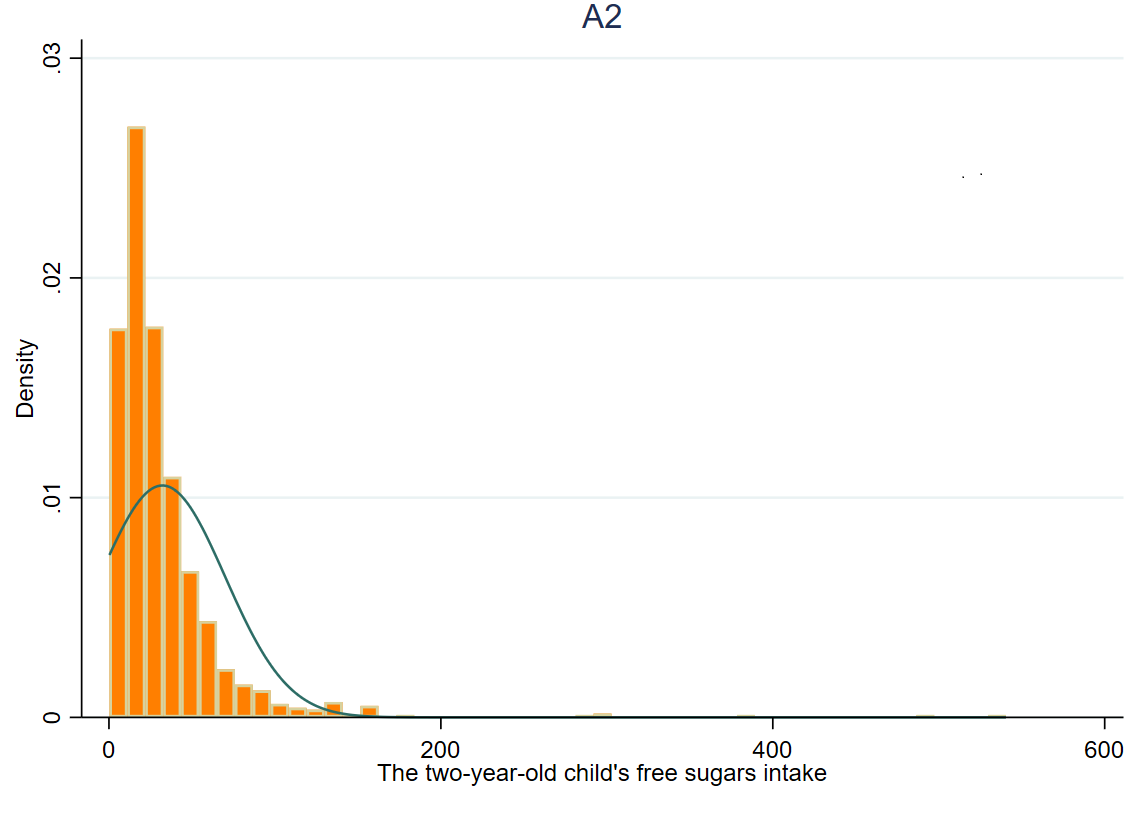 | 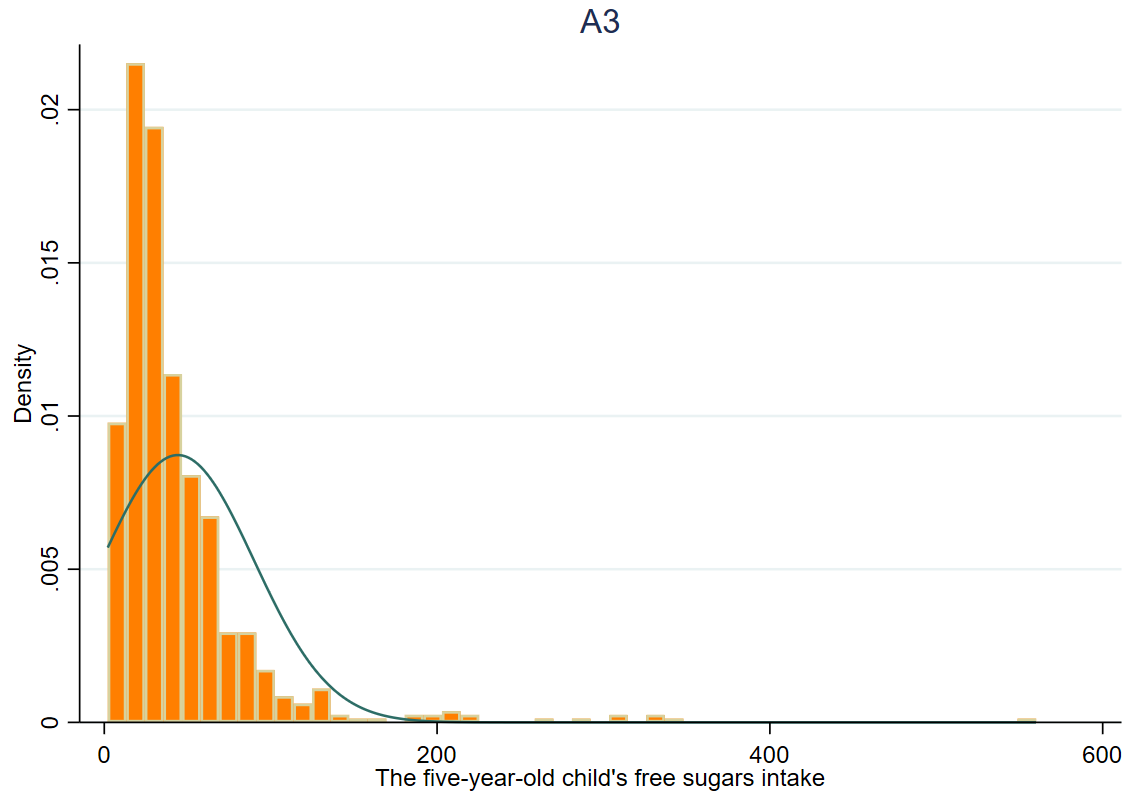 |
| --- | --- | --- |
| 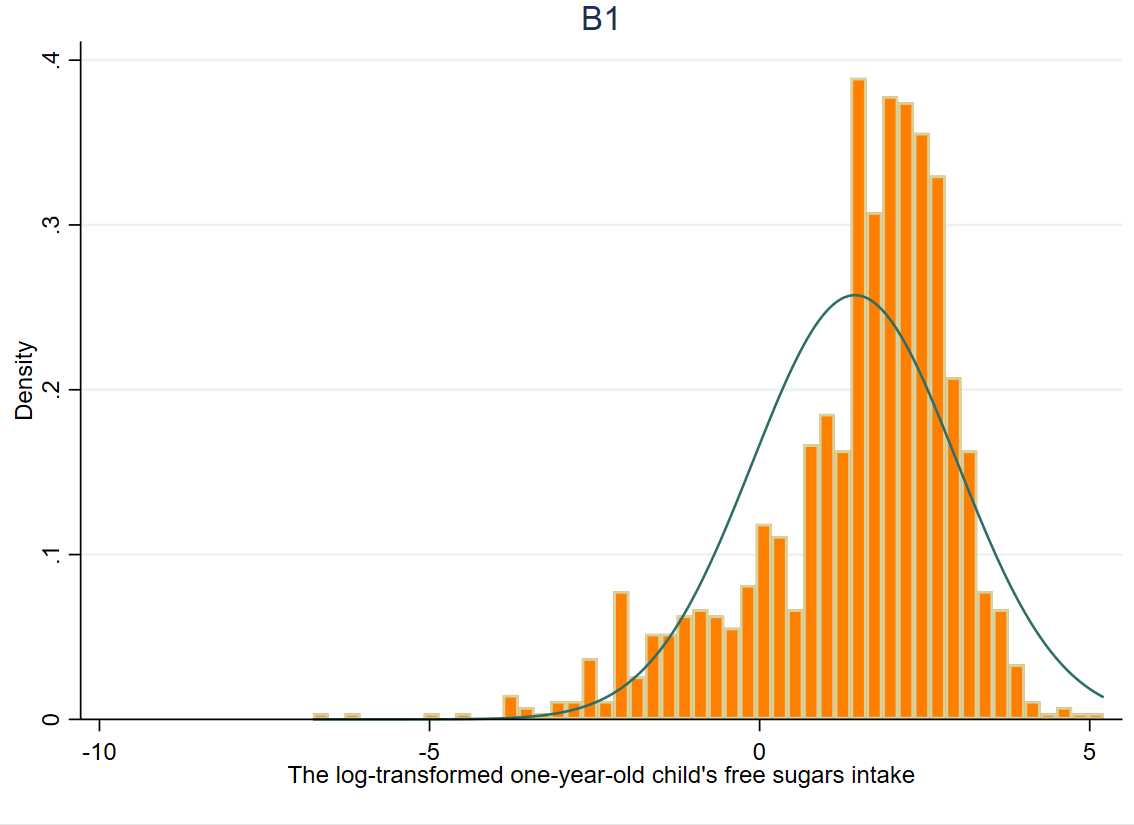 | 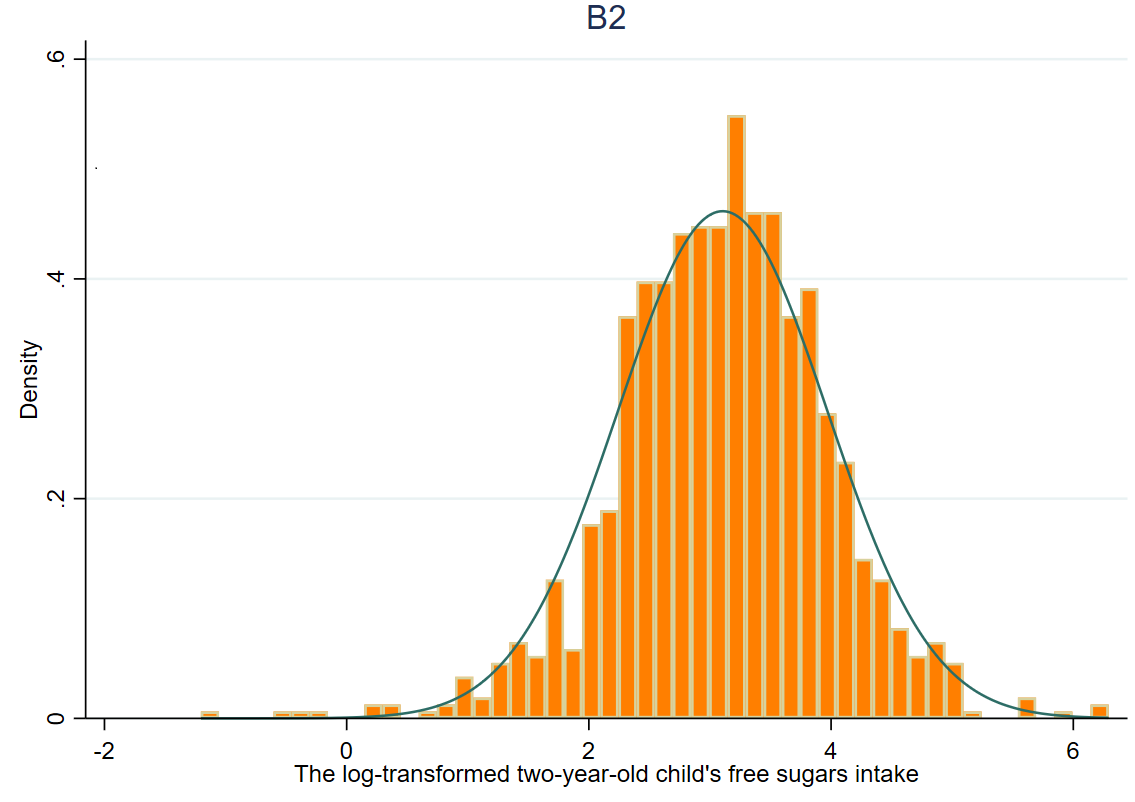 | 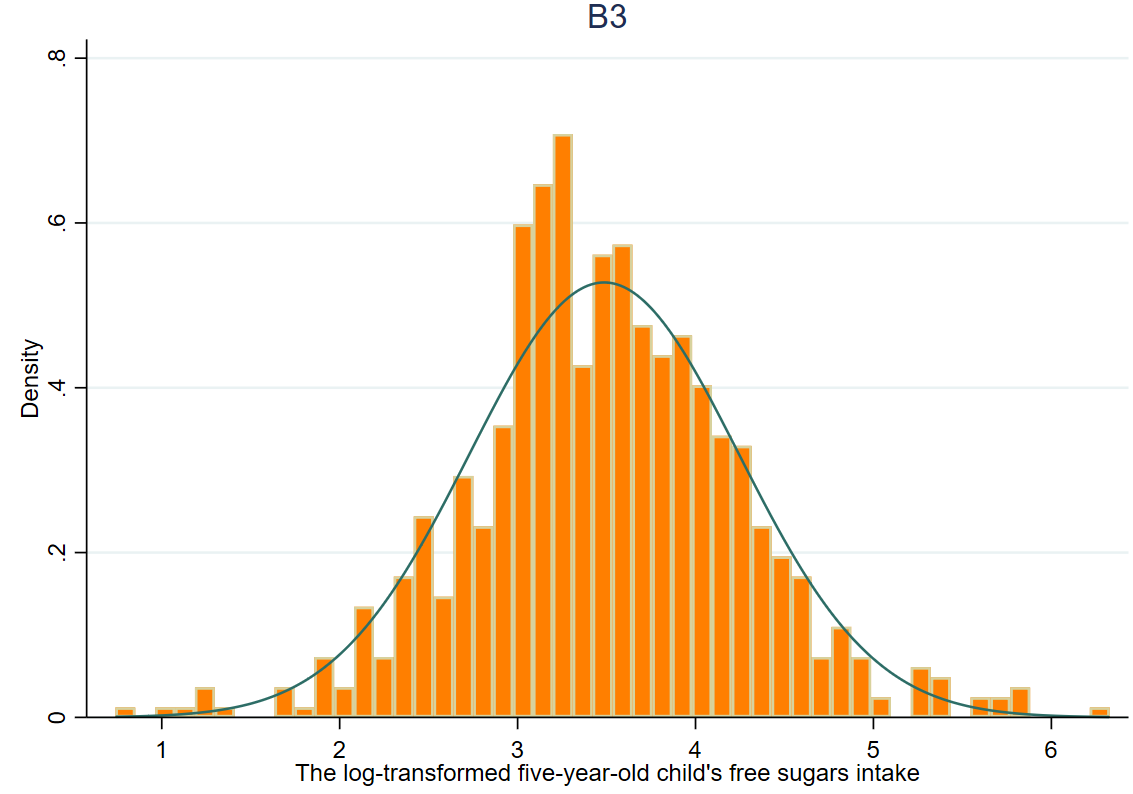 |

**Supplementary table 1. The single trajectory quadratic model for censored normal distribution of three waves of child’s free sugars intake**

| **Group Parameter** | **Estimate** | **SE** | **T for H0: Parameter=0** | ***p*-value** |
| --- | --- | --- | --- | --- |
| 1 Intercept | -1.50 | 0.15 | -9.67 | 0.000 |
| Linear | 3.58 | 0.18 | 20.06 | 0.000 |
| Quadratic | -0.64 | 0.05 | -14.22 | 0.000 |
|  |  |  |  |  |
| Sigma | 1.16 | 0.02 | 76.33 | 0.000 |
|  |  |  |  |  |
| **Group membership** |  |  |  |  |
| 1 (%) | 100 | 0.00 |  |  |

BIC= -4579.25 (N=1386), AIC= -4568.78, Log-likelihood= -4564.78

**Supplementary figure 2. Trajectories of the single-trajectory quadratic model of child’s free sugars intake**

**Supplementary table 2. The two-trajectory quadratic model for censored normal distribution of three waves of child’s free sugars intake**

| **Group Parameter** | **Estimate** | **SE** | **T for H0: Parameter=0** | ***p*-value** |
| --- | --- | --- | --- | --- |
| 1 Intercept | -8.84 | 0.36 | -24.44 | 0.000 |
| Linear | 9.27 | 0.40 | 23.34 | 0.000 |
| Quadratic | -1.76 | 0.10 | -18.11 | 0.000 |
|  |  |  |  |  |
| 2 Intercept | -0.07 | 0.13 | -0.54 | 0.593 |
| Linear | 2.47 | 0.15 | 16.25 | 0.000 |
| Quadratic | -0.42 | 0.04 | -11.09 | 0.000 |
|  |  |  |  |  |
| Sigma | 0.86 | 0.01 | 71.60 | 0.000 |
|  |  |  |  |  |
| **Group membership** |  |  |  |  |
| 1 (%) | 16.23 | 1.29 | 12.57 | 0.000 |
| 2 (%) | 83.77 | 1.29 | 64.91 | 0.000 |

BIC= -4173.91 (N=1386), AIC= -4152.98, Log-likelihood= -4144.98

**Supplementary figure 3. Trajectories of the two-trajectory quadratic model of child’s free sugars intake**

**Supplementary table 3. The three-trajectory quadratic model for censored normal distribution of three waves of child’s free sugars intake**

| **Group Parameter** | **Estimate** | **SE** | **T for H0: Parameter=0** | ***p*-value** |
| --- | --- | --- | --- | --- |
| 1 Intercept | -9.97 | 0.47 | -21.20 | 0.000 |
| Linear | 10.29 | 0.47 | 21.69 | 0.000 |
| Quadratic | -1.97 | 0.11 | -17.66 | 0.000 |
|  |  |  |  |  |
| 2 Intercept | -1.42 | 0.71 | -2.01 | 0.045 |
| Linear | 3.04 | 0.56 | 5.39 | 0.000 |
| Quadratic | -0.51 | 0.12 | -4.35 | 0.000 |
|  |  |  |  |  |
| 3 Intercept | 0.34 | 0.17 | 1.98 | 0.048 |
| Linear | 2.35 | 0.19 | 12.49 | 0.000 |
|  | -0.41 | 0.05 | -8.77 | 0.000 |
| Sigma |  |  |  |  |
|  |  |  |  |  |
| **Group membership** |  |  |  |  |
| 1 (%) | 12.75 | 1.58 | 8.06 | 0.000 |
| 2 (%) | 28.44 | 7.90 | 3.60 | 0.000 |
| 3 (%) | 58.81 | 8.84 | 6.65 | 0.000 |

BIC= -4147.97 (N=1386), AIC= -4116.56, Log-likelihood= -4104.56

**Supplementary figure 4. Trajectories of the three-trajectory quadratic model of child’s free sugars intake**

**Supplementary table 4. The four-trajectory quadratic model for censored normal distribution of three waves of child’s free sugars intake**

| **Group Parameter** | **Estimate** | **SE** | **T for H0: Parameter=0** | ***p*-value** |
| --- | --- | --- | --- | --- |
| 1 Intercept | -15.17 | 1.28 | -11.85 | 0.000 |
| Linear | 13.93 | 1.39 | 10.00 | 0.000 |
| Quadratic | -2.60 | 0.33 | -7.82 | 0.000 |
|  |  |  |  |  |
| 2 Intercept | -8.39 | 0.39 | -21.29 | 0.000 |
| Linear | 9.07 | 0.41 | 22.39 | 0.000 |
| Quadratic | -1.73 | 0.10 | -17.92 | 0.000 |
|  |  |  |  |  |
| 3 Intercept | 0.36 | 0.20 | 1.81 | 0.070 |
| Linear | 2.51 | 0.22 | 11.44 | 0.000 |
| Quadratic | -0.44 | 0.05 | -8.21 | 0.000 |
|  |  |  |  |  |
| 4 Intercept | -0.26 | 0.25 | -1.05 | 0.296 |
| Linear | 2.20 | 0.25 | 8.76 | 0.000 |
| Quadratic | -0.35 | 0.06 | -5.86 | 0.000 |
|  |  |  |  |  |
| Sigma | 0.75 | 0.01 | 59.29 | 0.000 |
|  |  |  |  |  |
| **Group membership** |  |  |  |  |
| 1 (%) | 1.43 | 0.52 | 2.78 | 0.006 |
| 2 (%) | 15.37 | 1.34 | 11.43 | 0.000 |
| 3 (%) | 41.55 | 6.62 | 6.27 | 0.000 |
| 4 (%) | 41.66 | 6.38 | 6.53 | 0.000 |

BIC= -4117.20 (N=1386), AIC= -4075.33, Log-likelihood= -4059.33

**Supplementary figure 5. Trajectories of the four-trajectory quadratic model of child’s free sugars intake**

**Supplementary table 5. The three-trajectory (two quadratic, one linear) model for censored normal distribution of three waves of child’s free sugars intake**

| **Group Parameter** | **Estimate** | **SE** | **T for H0: Parameter=0** | ***p*-value** |
| --- | --- | --- | --- | --- |
| 1 Intercept | -9.21 | 0.37 | -25.03 | 0.0000 |
| Linear | 9.60 | 0.40 | 23.91 | 0.0000 |
| Quadratic | -1.82 | 0.10 | -18.70 | 0.0000 |
|  |  |  |  |  |
| 2 Intercept | -0.40 | 0.19 | -2.08 | 0.0376 |
| Linear | 2.62 | 0.18 | 14.21 | 0.0000 |
| Quadratic | -0.45 | 0.04 | -10.16 | 0.0000 |
|  |  |  |  |  |
| 3 Intercept | 2.11 | 0.16 | 13.56 | 0.0000 |
| Linear | 0.74 | 0.07 | 10.83 | 0.0000 |
|  |  |  |  |  |
| Sigma | 0.81 | 0.01 | 57.46 | 0.0000 |
|  |  |  |  |  |
| **Group membership** |  |  |  |  |
| 1 (%) | 15.14 | 1.28 | 11.85 | 0.0000 |
| 2 (%) | 68.29 | 5.29 | 12.91 | 0.0000 |
| 3 (%) | 16.57 | 5.50 | 3.01 | 0.0026 |

BIC= -4166.15 (N=1386), AIC= -4137.36, Log-likelihood = -4126.36

**Supplementary figure 6. Trajectories of the three-trajectory quadratic and linear model of child’s free sugars intake**

| 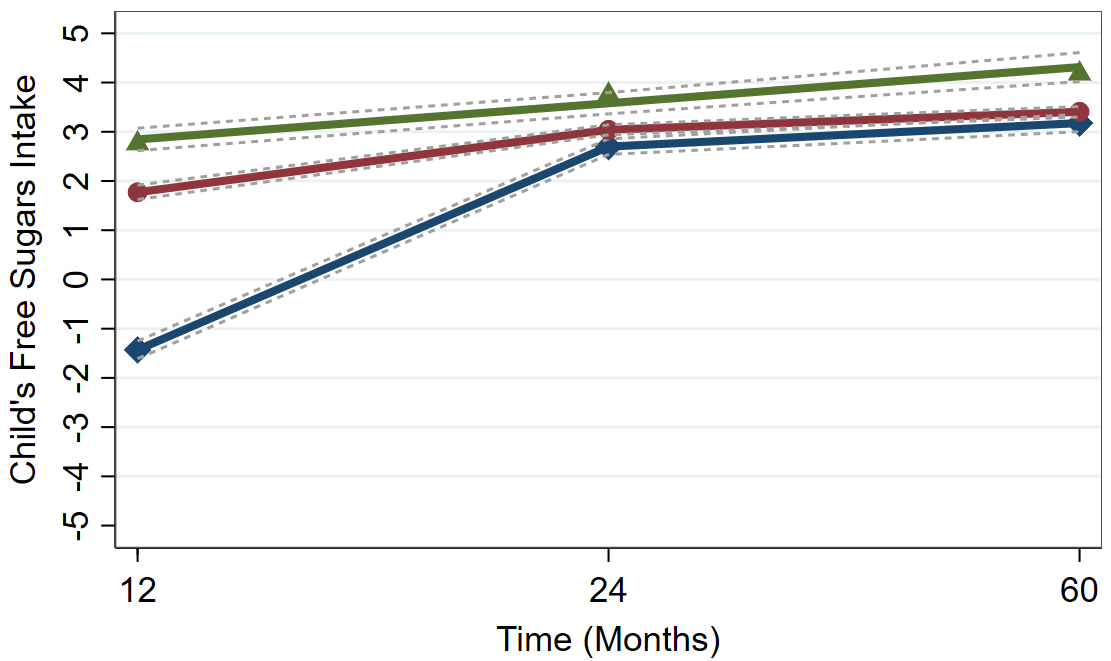 |
| --- |
| \|  \| Low fast increasing (15.1%) \| \| --- \| --- \| \|  \| Moderate increasing (68.3%) \| \|  \| High increasing (16.6%) \| |

**Supplementary table 6. A comparison of BIC and the log Bayes factor across the single-, two-, three-, and four-trajectory quadratic model of three waves of child’s free sugars intake**

| **Model type** | **BIC** | **Log Bayes factor** |
| --- | --- | --- |
| 1. Single quadratic | -4579.25 | reference |
| 2. Two-trajectory quadratic | -4173.91 | 810.68^a^ |
| 3. Three-trajectory quadratic | -4147.97 | 51.88^b^ |
| 4. Four-trajectory quadratic | -4117.20 | 61.54^c^ |
| 5. Three-trajectory model (two-quadratic and one-linear) | -4166.15 | 15.52^d^ |

^a^The log Bayes factor of model 2 vs. model 1

^b^The log Bayes factor of model 3 vs. model 2;

^c^The log Bayes factor of model 4 vs. model 3;

^d^The log Bayes factor of model 5 vs. model 2.

**Supplementary table 3. A comparison of model fit between the three-trajectory quadratic model and the three-trajectory (two-quadratic and 1-linear) model of three waves of child’s free sugars intake**

| **Model type** | **Trajectory** | **Total observations** | **GMP (95%CI)** | **95%CI difference** | **AvePP** | **Weighted OCC** | **EP** |
| --- | --- | --- | --- | --- | --- | --- | --- |
| Three-trajectory quadratic |  |  |  |  |  |  |  |
|  | 1 | 143 | 12.7 (9.6, 15.8) | 6.2 | 92.4 | 83.4 | 10.3 |
|  | 2 | 344 | 28.4 (12.9, 43.9) | 31.0 | 69.5 | 5.7 | 24.7 |
|  | 3 | 908 | 59.0 (41.4, 76.1) | 34.7 | 81.4 | 3.0 | 65.0 |
| Three-trajectory model (two-quadratic and one-linear) |  |  |  |  |  |  |  |
|  | 1 | 165 | 15.1 (12.6, 17.6) | 5.0 | 94.0 | 85.4 | 11.8 |
|  | 2 | 1104 | 68.3 (58.0, 78.6) | 20.6 | 81.7 | 2.1 | 79.1 |
|  | 3 | 126 | 16.6 (5.8, 27.3) | 21.5 | 68.0 | 10.6 | 9.0 |

GMP: Group membership probabilities (or group proportions based on posterior probabilities)

AvePP: Average posterior probabilities

OCC: Odds of correct classification

EP: Estimated probabilities (or Predicted probabilities)

95%CI difference: The upper limit – the lower limit

**Supplementary figure 7. Observed free sugars intake individual lines**


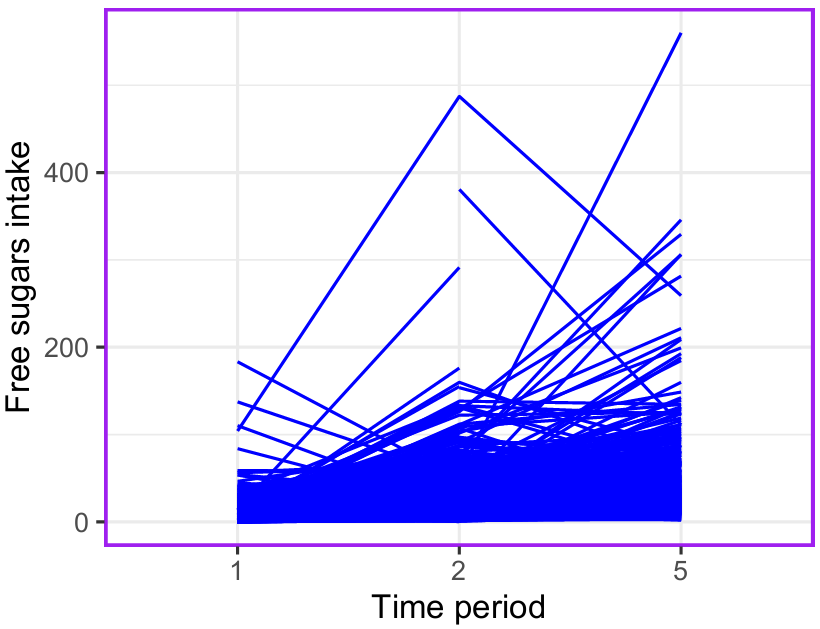


**References**

1. Nagin, D.S., *Group-Based Modeling of Development*. 2005: Harvard University Press, Cambridge, MA.

2. Andruff, H., et al., *Latent Class Growth Modelling: A Tutorial.* Tutorials in Quantitative Methods for Psychology, 2009. **5**(1): p. 11‐24.

3. Jones, B.L., D.S. Nagin, and K. Roeder, *A SAS procedure based on mixture models for estimating developmental trajectories.* Sociological Methods and Research, 2001. **29**: p. 374‐393.
